# Somatic mutations in tumor and plasma of locoregional recurrent and/or metastatic head and neck cancer using a next‐generation sequencing panel: A preliminary study

**DOI:** 10.1002/cam4.5436

**Published:** 2022-11-24

**Authors:** Óscar Rapado‐González, Jenifer Brea‐Iglesias, Aitor Rodríguez‐Casanova, Aida Bao‐Caamano, José‐Luis López‐Cedrún, Gabriel Triana‐Martínez, Roberto Díaz‐Peña, María Arminda Santos, Rafael López‐López, Laura Muinelo‐Romay, Mónica Martínez‐Fernández, Ángel Díaz‐Lagares, María Mercedes Suárez‐Cunqueiro

**Affiliations:** ^1^ Department of Surgery and Medical‐Surgical Specialties, Medicine and Dentistry School Universidade de Santiago de Compostela (USC) Santiago de Compostela Spain; ^2^ Galician Precision Oncology Research Group (ONCOGAL), Medicine and Dentistry School Universidade de Santiago de Compostela (USC) Santiago de Compostela Spain; ^3^ Liquid Biopsy Analysis Unit, Translational Medical Oncology Group (ONCOMET) Health Research Institute of Santiago (IDIS) Santiago de Compostela Spain; ^4^ Centro de Investigación Biomédica en Red en Cáncer (CIBERONC) Instituto de Salud Carlos III Madrid Spain; ^5^ Translational Molecular Oncology Unit, Galicia Sur Health Research Institute (IIS Galicia Sur) SERGAS‐UVIGO, Hospital Álvaro Cunqueiro Vigo Spain; ^6^ Epigenomics Unit, Cancer Epigenomics, Translational Medical Oncology Group (ONCOMET) Health Research Institute of Santiago (IDIS), Complexo Hospitalario Universitario de Santiago de Compostela (CHUS,SERGAS) Santiago de Compostela Spain; ^7^ Roche‐Chus Joint Unit, Translational Medical Oncology Group (ONCOMET) Health Research Institute of Santiago (IDIS) Santiago de Compostela Spain; ^8^ Universidade de Santiago de Compostela (USC) Santiago de Compostela Spain; ^9^ Department of Oral and Maxillofacial Surgery Complexo Hospitalario Universitario de A Coruña (CHUAC, SERGAS) A Coruña Spain; ^10^ Department of Radiation Oncology Centro Oncológico de Galicia A Coruña Spain; ^11^ Fundación Pública Galega de Medicina Xenómica, SERGAS, Grupo de Medicina Xenómica‐USC Health Research Institute of Santiago de Compostela (IDIS) Santiago de Compostela Spain; ^12^ Faculty of Health Sciences Universidad Autónoma de Chile Talca Chile; ^13^ Department of Oral Rehabilitation Instituto Universitario de Ciências da Saúde (IUCS) Gandra Portugal; ^14^ Translational Medical Oncology Group (ONCOMET) Health Research Institute of Santiago (IDIS), Complexo Hospitalario Universitario de Santiago de Compostela (CHUS, SERGAS) Santiago de Compostela Spain

**Keywords:** cell‐free DNA, droplet digital PCR, head and neck squamous cell carcinoma, liquid biopsy, next‐generation sequencing, saliva, somatic mutations

## Abstract

**Background:**

We explore the utility of TruSight Tumor 170 panel (TST170) for detecting somatic mutations in tumor and cfDNA from locoregional recurrent and/or metastatic head and neck squamous cell carcinoma (HNSCC).

**Methods:**

Targeted NGS of tumor DNA and plasma cfDNA was performed using TST170 panel. In addition, a set of somatic mutations previously described in HNSCC were selected for validating in tumor, plasma, and saliva by digital droplet PCR.

**Results:**

The TST170 panel identified 13 non‐synonymous somatic mutations, of which five were detected in tumoral tissue, other five in plasma cfDNA, and three in both tissue and plasma cfDNA. Of the eight somatic mutations identified in tissue, three were also identified in plasma cfDNA, showing an overall concordance rate of 37.5%.

**Conclusions:**

This preliminary study shows the possibility to detect somatic mutations in tumor and plasma of HNSCC patients using a single assay that would facilitate the clinical implementation of personalized medicine in the clinic.

## Graphical abstract

This study aims to explore whether a NGS panel (TST170) could be suitable for detecting somatic mutations in tumor and cfDNA from locoregional recurrent and/or metastatic HNSCC patients. Furthermore, we also carried out an orthogonal validation of somatic mutations in tumor, plasma cfDNA, and salivary DNA.

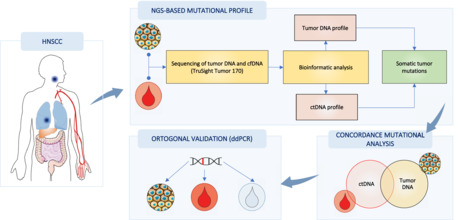

## 1 INTRODUCTION

Head and neck squamous cell carcinoma (HNSCC) is a malignancy characterized by heterogeneous nature both at clinical and molecular points of view. Despite advances in diagnosis and multimodal treatment strategies, 65% of HNSCC patients develop recurrent or metastatic disease, associated with an unfavorable prognosis.^1^ The characterization of the molecular landscape of HNSCC has revealed several driver genes involved in head and neck carcinogenesis, such as *TP53*, *CDKN2A*, *PIK3CA*, *NOTCH1*, *FAT1*, or *EGFR*, representing an opportunity for developing molecular targeted therapies. Furthermore, molecular subtyping has shown different genetic alterations in human papillomavirus (HPV) negative head and neck cancer (HNC), which are mainly characterized by the accumulation of mutations in *TP53* and *CDKN2A* tumor suppressor genes.^2^ Nowadays, the epidermal growth factor receptor (*EGFR*) is the only proven molecular target for HNC management, representing an alternative therapy to cisplatin unfit patients.^3^ Unfortunately, still today the standard of care in HNC is based on the clinical and histopathologic characteristics of the tumor, so there is a great need to identify real‐time tumor biomarkers for guiding therapy selection in HNC that will help to improve the therapeutic response rates in these patients.

Liquid biopsies based on circulating tumor DNA (ctDNA), a fraction of total cell‐free DNA (cfDNA), have emerged as a potential non‐invasive approach for tumoral genome profiling.^4^ Ongoing advances in next‐generation sequencing (NGS) technology have allowed the development of multiple‐gene panels for characterizing tumors in both tissue and cfDNA samples. The feasibility to identify tumor somatic genomic alterations such as single nucleotide variants (SNVs), insertions and deletions (InDels), or copy number alterations (CNVs) in cfDNA, highlights its clinical value for targeted therapy‐decision making and monitoring treatment response in cancer.^5^, ^6^, ^7^ Due to the molecular landscape of HNSCC harbors potentially actionable alterations, cfDNA profiling could represent an alternative to tumor biopsy for detecting and monitoring somatic alterations over the course of the disease, which will allow the application of precision medicine in HNC. Recently, some studies have concentrated on the identification of somatic mutations in liquid biopsies using a tumor‐tissue informative approach^8^, ^9^ or a tumor‐agnostic approach.^10^, ^11^ This pilot study aims to explore whether a NGS panel (TST170) could be suitable for detecting somatic mutations in tumor and cfDNA from locoregional recurrent and/or metastatic HNSCC patients. Furthermore, we also carried out an orthogonal validation of somatic mutations in tumor, plasma cfDNA, and salivary DNA.

## 2 MATERIALS AND METHODS

### 2.1 HNSCC patients' recruitment

This study was approved by the Research Ethics Committee Networks in Galicia (Ref. No. 2018/003) and carried out under the principles of the Helsinki Declaration (World Medical Association, 2013). Written consent was obtained for each patient for use of their tissues and liquid biopsies (blood and saliva) samples. This retrospective study enrolled patients diagnosed with locoregional recurrent and/or metastatic HPV‐negative HNSCC. Patients were diagnosed by anatomo‐pathologic analysis of the corresponding tissue biopsy and HPV‐status was determined using Anyplex II HPV28 (Seegene**®**) detection kit. HNSCC patients (*n* = 3) were obtained at Department of Oral and Maxillofacial Surgery from the Complexo Hospitalario Universitario of A Coruña (CHUAC, SERGAS) and at Department of Radiation Oncology, Centro Oncológico of Galicia (COG), in Galicia, Spain. Blood and saliva samples were collected at diagnosis of locoregional recurrent and/or metastatic disease and primary tumor samples were obtained of tissue biopsy specimens from biobank. Patient's anatomo‐pathologic data are shown in Table S1.

### 2.2 Plasma and saliva samples collection

Peripheral blood samples were collected by drawing blood into Streck Cell‐Free DNA BCT® tubes. A two‐step centrifugation was carried out to isolate the plasma. First, blood was centrifuged at 1600x g for 10 min at room temperature. Then, plasma was collected without disturbing the buffy coat layer and centrifuged at 5500x g for 10 min at room temperature to remove remaining cells. Plasma samples were stored at −80°C for further analysis.

Salivary samples were collected using the Oragene® DNA Sample Collection Kit (DNA Genotek OG‐500; Ottawa, Ontario, Canada) according to the manufacturer's instructions. Then, saliva samples were stored at −80°C until further analysis.

### 2.3 DNA extraction

Genomic DNA was isolated from formalin‐fixed paraffin‐embedded (FFPE) tissues using the AllPrep® DNA/RNA FFPE kit (Qiagen) according to the manufacturer's recommendations. FFPE samples contained at least 40% tumor cells on the hematoxylin and eosin slide. Genomic DNA from tumor samples was eluted in 100 μl elution buffer AE and stored at −20°C for further use. DNA from saliva and plasma cfDNA was isolated using the QIAamp DNA Circulating Nucleic Acid Kit (Qiagen), according to the manufacturer's instructions. Samples were eluted in 100 μl of buffer AVE and stored at −20°C until further use. Isolated DNA from tumor, plasma, and saliva was quantified by Qubit 4.0 Fluorometer (Thermo Fisher Scientific). Agilent's TapeStation 4200 (Agilent Technologies) was used to assess the fragment distribution of the extracted DNA.

### 2.4 NGS‐based mutational analysis

Targeted NGS sequencing of genomic DNA and plasma cfDNA was performed using the Illumina TruSight Tumor 170 panel (TST170). This hybrid‐capture panel is designed to target DNA variants of 170 cancer‐related genes. The panel includes 55 genes for fusions and splice variants, 148 SNVs and InDels, and 59 amplifications (Table S2). A total of 60 ng of genomic DNA (FFPE samples) or cfDNA (plasma samples) were used as input for sequencing library preparation. Following manufacturer's protocol, DNA from FFPE samples was first fragmented with an ultrasonocator (Covaris S220). This fragmentation step was omitted in the case of cfDNA samples. All the next steps were performed following the manufacturer's reference guides. A pool of oligos specific to the 170 genes was hybridized to DNA libraries for a minimum of 8 h to a maximum of 24 h at 57°C. To ensure high specificity of the captured regions, a second hybridization step was performed for a minimum of 1.5 h to a maximum of 4 h at 57°C. After amplification of the captured regions, the quantity of enriched libraries was assessed using a fluorometric method (Qubit) and, further, a bead‐based normalization was carried out. Sequencing was carried out on NextSeq 500 sequencing system (Illumina), according to the TruSight® Tumor 170 protocol (Illumina).

### 2.5 Bioinformatic analysis

FASTQ sequencing reads were assessed using the quality control tool, FastQC.^12^ Reads were mapped to the hg19 reference genome by Burrows–Wheeler Aligner (BWA)‐MEM.^13^ SAMtools were used to convert the aligned SAM files to BAM files,^14^ applying Bammarkduplicates2 to mark duplicated reads.^15^ mosDepth was used for quantifying coverage from BAM input files (https://github.com/brentp/mosdepth). Variant calling was performed using the Genome Analysis Tool Kit (GATK),^16^ together with the Mutect2 tool in GATK (Benjamin D et al. 2019; https://www.biorxiv.org/content/10.1101/861054v1), following best practices (https://gatk.broadinstitute.org/hc/en‐us/articles/360035894731‐Somatic‐short‐variant‐discovery‐SNVs‐Indels‐). The Ensembl Variant Effect Predictor (VEP) was applied to determine the effect of genetic variants,^17^ using SIFT and Polyphen scores to establish the effect of differently called genetic variants.^18^, ^19^ Following VEP annotations, variants were filtered in order to preserve just those classified as probably pathogenic both by SIFT and Polyphen, as well as those with a high impact. As an additional control for somatic variants, each of them must present an allele frequency observed in any population from 1KGP, ESP, or gnomAD lower than 0.01 or not described, to assess they are not common in the population.^20^

### 2.6 Validation of somatic mutations by digital droplet PCR

Somatic mutations were detected by digital droplet PCR (ddPCR) (Bio‐Rad Laboratories), using the QX200 ddPCR system (Bio‐Rad Laboratories). Data analysis was performed on the QuantaSoftTM v.1.7 software (Bio‐Rad Laboratories) according to manufacturer's recommendations.

## 3 RESULTS

### 3.1 Analysis of somatic gene alterations in tumor and plasma cfDNA of HNSCC patients by TST170

**FIGURE 1:**
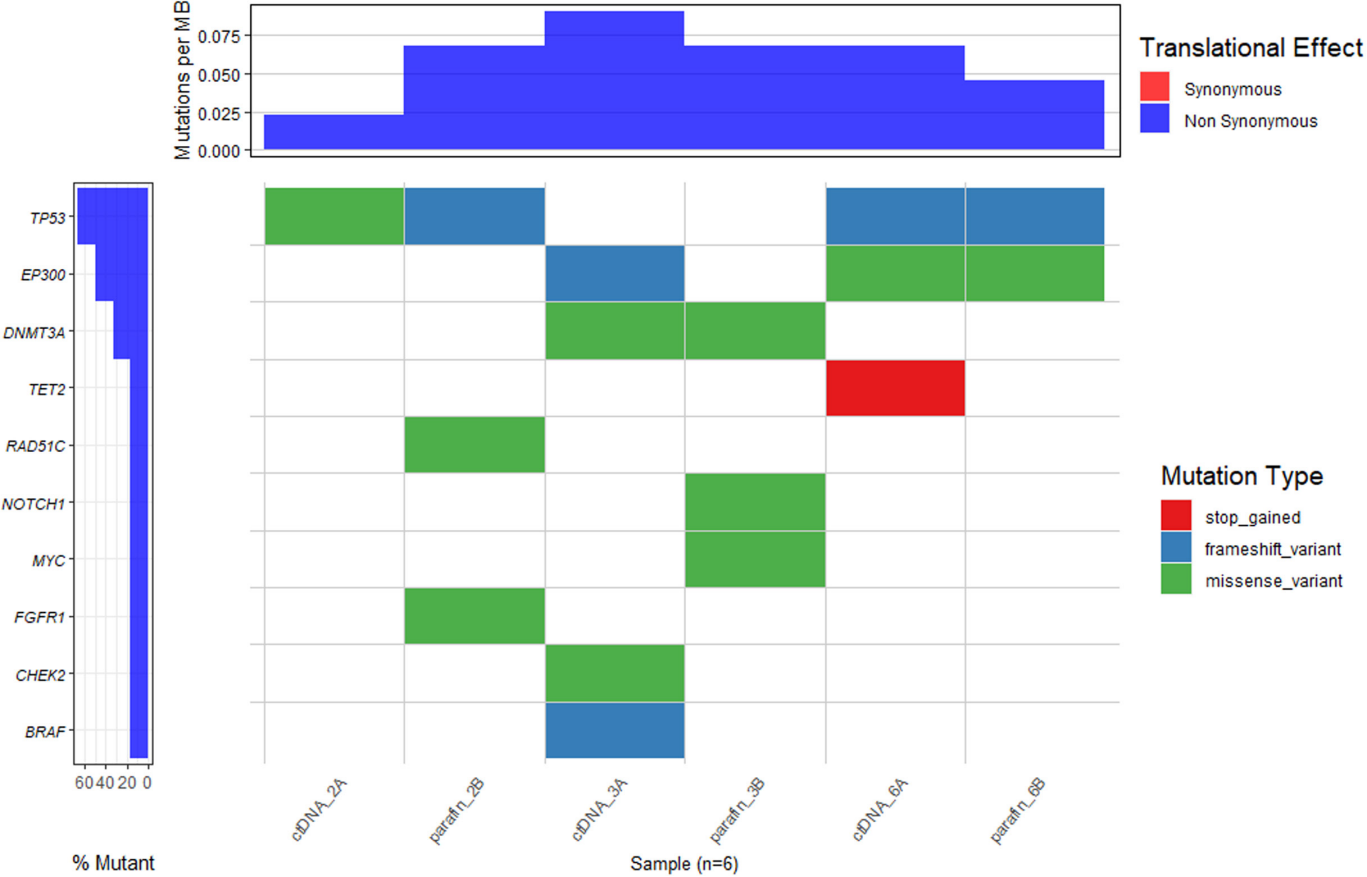
Matrix of somatic mutations detected in tumor and plasma cfDNA of the three advanced HNSCC patients.

Targeting DNA sequencing using the TST170 panel identified 13 non‐synonymous somatic mutations in tumoral tissue and/or plasma cfDNA of 3 patients (Figure 1). Overall, the detected somatic gene alterations correspond to missense (61.53%), frameshifts (30.76%), and stop gain (7.69%) variants. Interestingly, we found in tumor samples three somatic mutations in patient No. 3, three in patient No. 2, and two in patient No. 6. The targeted NGS of tumoral DNA by TST170 provided a mean coverage of 1120x (range: 15x − 4937x). The allelic frequency of mutant copies ranged from 1.1–50.6 in tumor samples (Table S3). The targeted NGS of cfDNA by TST170 provided a mean coverage of 2008x (range: 4x − 3837x). The allelic frequency mutant copies ranged from 1.1–5.4 in plasma samples. Of the eight somatic mutations identified in tissue, three were also identified in plasma cfDNA, showing an overall concordance rate of 37.5%. A total of eight somatic variants were identified in plasma, of which five (62.5%) were not identified in tumor tissue.

Analyzing the intra‐patient concordance, 1/6 variants in patient No. 3 (16.66%), and 2/3 (66.66%) in patient No. 6 were found in both tumor and plasma.

### 3.2 Analysis of selected somatic mutations in tumor, plasma cfDNA, and salivary DNA of HNSCC patients by ddPCR

**FIGURE 2:**
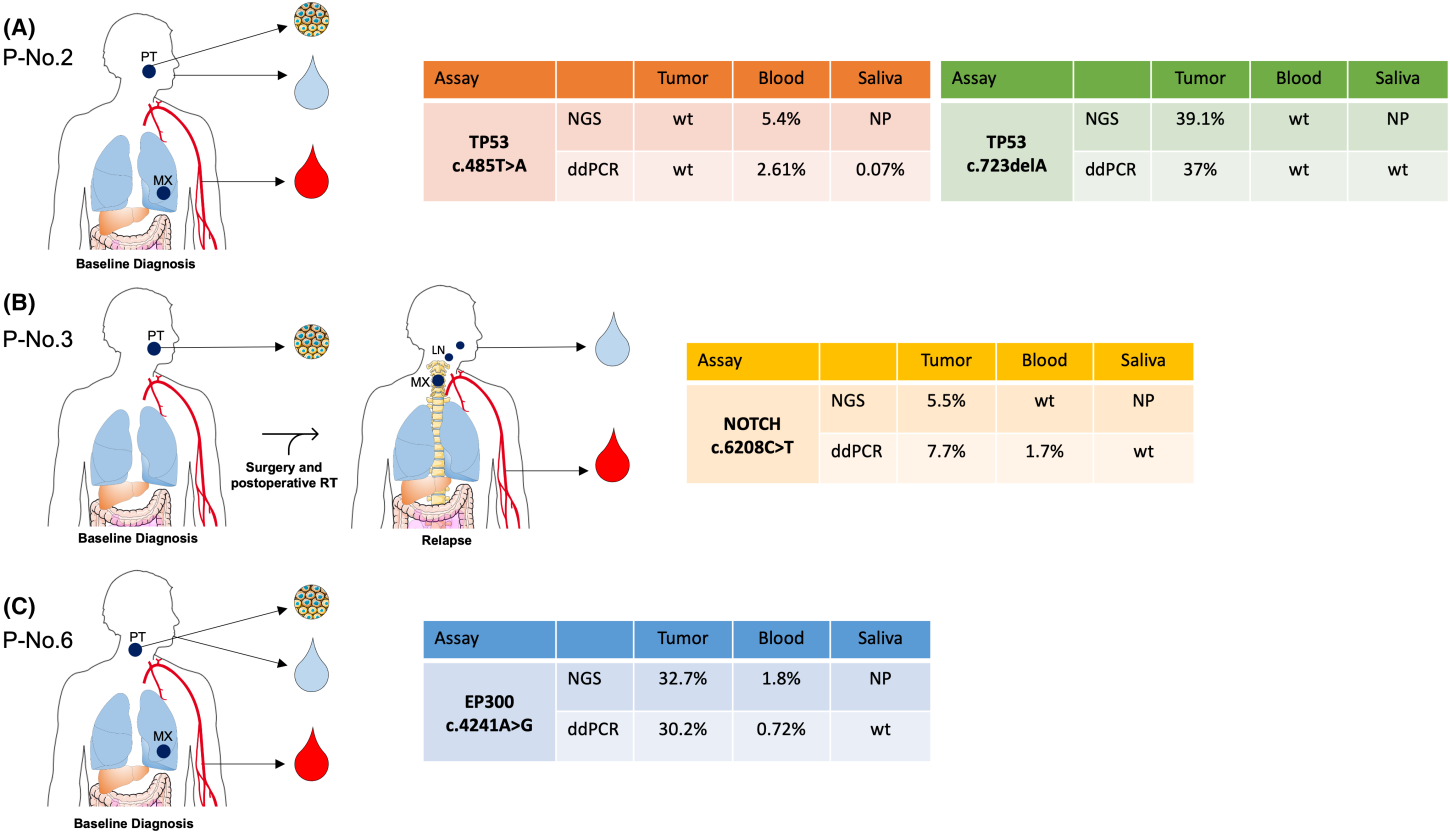
Validation of selected somatic mutations detected by NGS (TST 170 panel) on tissue, plasma, and saliva from HNSCC patients by ddPCR. (A) Patient No. 2 with tongue squamous cell carcinoma and lung metastasis (T3N2M1) at baseline diagnosis; (B) Patient No. 3 with gingival squamous cell carcinoma at baseline diagnosis and after surgery and postoperative radiotherapy (RT) treatment, locoregional lymph node recurrence, and distant metastasis in vertebra (T4N3M1); and (C) Patient No. 6 with hypopharynx tumor and lung metastasis at baseline diagnosis (T3N0M1). ddPCR, digital droplet PCR; LN, lymph nodes; MX, metastasis; NGS, next‐generation sequencing; NP, not performed; PT, primary tumor; WT, wild‐type.

A total of four somatic genetic alterations previously described in HNC (*EP300* c.4241A > G, *NOTCH1* c.6208C > T, *TP53* c.723delA, and *TP53* c.485 T > A) were selected to orthogonally validate our NGS analysis using ddPCR. In addition to tumor tissue and plasma cfDNA, we included paired salivary DNA from the three HNSCC patients. The *EP300* c.4241A > G mutation was validated in tumor DNA and plasma cfDNA with an allele frequency of mutant copies of 30.2% and 0.72%, respectively (Patient No. 6). The *NOTCH1* c.6208C > T mutation, detected only in tumor DNA by NGS was validated in tumor and plasma samples with an allele frequency of mutant copies of 7.7% and 1.7%, respectively (Patient No. 3). The *TP53* c.723delA mutation, detected in the tumor by NGS, was validated in tumor DNA with an allele frequency of mutant copies of 37% (Patient No. 2). The *TP53* c.485 T > A detected in plasma by NGS was validated by ddPCR in plasma and saliva samples with an allele frequency of mutant copies of 2.61% and 0.07%, respectively (Patient No. 2) (Figure 2; Table S4).

## 4 DISCUSSION

Our pilot study showed the ability to detect somatic mutations in cfDNA from HNSCC patients using the TST170 targeted NGS panel designed for screening genetic alterations in solid tumors. Recently, our research group demonstrated the potential clinical utility of the TST170 panel for detecting gene variants in cfDNA from metastatic colorectal cancer patients, supporting the application of this NGS approach to non‐invasively characterizing the genetic landscape of tumors.^21^ In the present study, we evaluate for the first time the applicability of the TST170 panel on tumor tissue and cfDNA from HNSCC patients. The molecular characterization of HNC patients using cfDNA has emerged as an opportunity for identifying predictive biomarkers and developing targeted therapies. We detected somatic variants (SNVs and InDels) in a total of ten genes in tumor and/or cfDNA, of which *TP53*, *NOTCH1*, and *EP300* are described as common driver genes in HPV‐negative HNC.

The feasibility of detecting somatic alterations in liquid biopsies from HNSCC has been previously explored in different studies.^11^, ^22^, ^23^ Wang et al. analyzed HPV and somatic mutations in *TP53*, *PI3KCA*, *CDKN2A*, *FBXW7*, *HRAS*, and *NRAS* in plasma cfDNA and salivary DNA of 93 HNSCC patients. Although a higher rate of ctDNA detection was observed in plasma for advanced stage disease and in saliva for early‐stage disease, this PCR approach was restricted to a limited set of genes previously identified in tumor biopsy.^22^ Later, Perdomo et al. analyzed by ctDNA sequencing 65 tumor mutations in 5 genes (*TP53*, *NOTCH1*, *CDKN2A*, *CASP8*, and *PTEN*) observing ctDNA alterations in 42% of plasma samples, with an overall concordance rate of 28%.^23^ Using a more comprehensive genomic approach, we explored the performance of TST170 panel in detecting gene variants in plasma cfDNA and tumor DNA from HNSCC due to this panel covered 60% of the most common mutated tumor suppressor genes and oncogenes in HNC based on COSMIC database. An overall concordance rate of 37.5% was found between plasma cfDNA and tumoral tissue in advanced HNSCC patients. Similarly, Galot et al. sequenced both cfDNA from plasma and tumor DNA by a custom panel of 604 genes in 39 HNSCC patients showing a concordance rate of 42% in metastatic HNC patients. However, this rate decreased to 18% in patients with locally advanced disease.^11^ Interestingly, lower concordance rates have been reported when different clinically available NGS panels were applied for sequencing tumor tissue and cfDNA.^24^, ^25^ To overcome this challenge, we tested the applicability of a targeted NGS tumor panel for detecting somatic alterations in cfDNA. The use of targeted NGS panels that allows the detection of gene variants in tumor tissue and cfDNA at the same time may provide a potential strategy for the molecular characterization of the tumor in a single assay without the need of designing a specific cfDNA panel. In addition, ctDNA sequencing at primary diagnosis can reflect the somatic gene alterations both primary tumor and metastatic lesions contributing to capture the mutational profile heterogeneity that may be underestimated by tissue biopsy.^26^, ^27^, ^28^ In this sense, 62.5% of the plasma variants were not found in tumor tissue that could be explained by the high intratumorally heterogeneity of HNC that is not represented in tissue biopsy samples. Therefore, sequencing of cfDNA can provide valuable information into tumor heterogeneity and clonal evolution from diagnosis through disease progression.

Our study included cfDNA from three advanced HNSCC patients. Of the three sequenced patients, liquid biopsies were taken simultaneously as tumor biopsy in two patients (No. 2 and No. 6), whereas in the third patient (No. 3) liquid biopsies were taken at the recurrence and tumor tissue biopsy at the time of primary disease. The patient No. 6 showed a high concordance rate (66.66%) between plasma cfDNA and tumor tissue biopsy. In this case, the tumor was located in the hypopharynx and the metastasis in the lung. Interestingly, the profiling of cfDNA revealed one somatic mutation in plasma that was not detected in tumoral tissue, providing molecular information about the metastatic profile of this patient. The patient No. 3 showed a concordance of 16.66% between cfDNA and tumor tissue. This lower concordance rate could be the result of the different moment of collection tumor biopsy (time of primary diagnosis) and liquid biopsies (at recurrence). In this case, the primary tumor was located at gingiva and the recurrence was in the cervical region with distant metastasis in vertebra. A total of three variants were detected exclusively in plasma probably reflecting the molecular evolution of the disease. However, in the patient No. 2, with a tongue tumor and lung metastasis any of somatic variants detected in tumor was identified in plasma. This discordance could be explained by the intratumor heterogeneity and specific metastatic profile.

Since saliva is the biofluid of the oral cavity, proximal and distant tumors may shed DNA into saliva representing an opportunity for detecting somatic mutations.^8^, ^29^ As tumor DNA was detected in saliva at frequencies <1%^8^, ^22^ we tested a set of gene variants reported as somatic mutations in HNSCC in total salivary DNA from our patient cohort by ddPCR. We were able to detect the mutation *TP53* c.485 T > A with a mutant allele fraction of 0.079% in saliva from patient No. 2 which confirms the possibility to detect somatic variants with an allele frequency <0.1% by ddPCR. As previously reported by Wang et al., the sensitivity for detecting tumor DNA in saliva was anatomic site‐dependent, showing a higher rate of detection and fraction of mutant alleles in oral cavity tumors compared to oropharynx, larynx, and hypopharynx tumors.^22^ Although in the other two cases (No. 3 and No. 6), tumor DNA was not detected in saliva, somatic mutations were validated in plasma. These findings suggest that plasma could be a more sensitive predictor for detecting tumor DNA than saliva in patients with advanced HNC.

One of the strengths of our work is the application of the same sequencing technology to tumor and cfDNA samples. TST170 allowed to detect somatic variations with a variant allele frequency <0.1% in ddPCR. Also, the detection of tumor‐specific mutations by ddPCR in liquid biopsies was technically feasible demonstrating the clinical applicability of this tumor‐tissue informative approach for detection and monitoring predictive biomarkers in HNC. However, our study has several limitations. The main limitation of our study is related to its sample size and the heterogeneous nature of this cohort. All patients included were male with advanced stages of HNSCC at different anatomic locations. This preliminary study was carried out to demonstrate the ability of the TST170 panel to detect somatic gene alterations in cfDNA samples of HNSCC patients. Another important limitation was that no tumoral tissue from metastatic sites was obtained. Also, the tumoral origin of the genetic variants detected exclusively in cfDNA should be considered cautiously due to the lack of data on peripheral blood leukocytes to explore the role of clonal hematopoiesis mutations. Although we corrected this issue by using a bioinformatic approach, our recommendation is to analyze matched germline DNA from each patient for identifying the contribution of clonal hematopoiesis to false positive detection. Furthermore, we only carried out the mutational analysis in plasma and saliva at a single time point which did not allow us to monitor mutations during treatment.

## 5 CONCLUSIONS

This preliminary study shows the possibility to detect somatic mutations in tumor and plasma of HNSCC patients using a single assay that would facilitate the clinical implementation of personalized oncology in the clinic. However, further research is necessary to validate our findings.

## AUTHOR CONTRIBUTIONS

**Oscar Rapado‐Gonzalez:** Investigation (equal); supervision (equal); validation (equal); visualization (equal); writing – original draft (equal); writing – review and editing (equal). **Jenifer Brea‐Iglesias:** Formal analysis (equal). **Aitor Rodriguez Casanova:** Investigation (equal). **Aida Bao‐Caamano:** Investigation (equal). **Jose Luis Lopez‐Cedrun:** Resources (equal). **Gabriel Triana‐Martinez:** Resources (equal). **Roberto Diaz‐Pena:** Visualization (equal). **María Arminda Santos:** Supervision (equal); visualization (equal). **Rafael Lopez‐Lopez:** Writing – review and editing (equal). **Laura Muinelo‐Romay:** Conceptualization (equal); methodology (equal); supervision (equal); validation (equal); writing – review and editing (equal). **Monica Martinez‐Fernandez:** Formal analysis (equal). **Angel Diaz‐Lagares:** Methodology (equal); writing – review and editing (equal). **Maria Suarez‐Cunqueiro:** Conceptualization (equal); funding acquisition (equal); methodology (equal); project administration (equal); supervision (equal); writing – original draft (equal); writing – review and editing (equal).

## FUNDING INFORMATION

The present investigation was funded by the Instituto de Salud Carlos III (ISCIII) and the European Regional Development Fund (FEDER) (PI20/01449), and Axencia Galega de Innovación (GAIN), Xunta de Galicia, Axudas do Programa de Consolidación e Estructuración de Unidades de Investigación Competitiva (IN607A2020/02).

## CONFLICT OF INTEREST

R.L.‐L. has received honoraria for participation in Advisory Boards from Roche, AstraZeneca, Merck, Merck Sharp & Dohme, Bayer, Bristol‐Myers Squibb, Novartis, Janssen, Lilly, Pfzer, and Leo; travel, accommodations, and expenses from PharmaMar, Roche, Bristol‐Myers Squibb, and Pierre Fabre; research funding from Roche and Merck; and is co‐founder and shareholder in Nasasbiotech, S.L., Mtrap Inc. The rest of the authors have nothing to disclose.

## ETHICS STATEMENT

This study was carried out according to the guidelines of the Helsinki Declaration and approved by the Research Ethics Committee Networks in Galicia (Ref. No. 2018/003).

## INFORMED CONSENT STATEMENT

Written informed consent was obtained from all participants.

## Supporting information


Table S1.
Table S2.
Table S3.
Table S4.

## Data Availability

The data that support the findings of this study are available from the corresponding author upon reasonable request.
