# Supplementary material for: Somatic mutations in tumor and plasma of locoregional recurrent and/or metastatic head and neck cancer using a next‐generation sequencing panel: A preliminary study
Source: Cancer Med. 2022 Nov 24;12(6):6615–22. doi: 10.1002/cam4.5436 (PMC10067107; doi:10.1002/cam4.5436)
Supplement: Supplementary file 1 — Table S1. Table S2. Table S3. Table S4. [file CAM4-12-6615-s001.docx]

**Table S1**. Clinicopathological characteristics of study subjects.

|  | **Patient No. 2** | **Patient No. 3** | **Patient No. 6** |
| --- | --- | --- | --- |
| **Age (years)** | 64 | 78 | 68 |
| **Gender** | Male | Male | Male |
| **Smoking status** | Current smoker  (>10 cig/day) | Non-smoker | Current smoker  (>10 cig/day) |
| **Alcohol intake** | Yes (Moderate) | Yes (Moderate) | Yes (Heavy) |
| **OPMD** | No | No | No |
| **Tumor location** | Tongue | Maxillary gingiva | Hypopharynx |
| **Tumor size** | 6 cm | 2.7 cm | 3 cm |
| **Macroscopic appearance** | Exophytic ulcer | Exophytic ulcer | Exophytic ulcer |
| **Lymph node levels involved (I/II/III/IV/V)** | II/III | III/IV | No |
| **Bilateral lymph node involvement (Yes/No)** | Yes | Yes | No |
| **Extracapsular spread (Yes/No)** | Yes | Yes | No |
| **Tumor recurrence** | - | Regional lymph nodes | - |
| **Metastasis Location** | Lung | Vertebra | Lung |
| **TNM Stage** | T3N2M1 | T0N3M1 | T3N0M1 |
| **Grade of differentiation** | G2 | G3 | G2 |
| **HPV-status** | Negative | Negative | Negative |
| **Survival time (months)** | 12 | 9 | 9 |

*Abbreviations:* OPMD, oral potentially malignant disorders; HPV, human papillomavirus; TNM, tumor-node-metastasis

**Table S2**. List of genes analyzed in DNA by the TST170 panel.

| SNV and InDels | | | | | | | | | |
| --- | --- | --- | --- | --- | --- | --- | --- | --- | --- |
| *AKT1* | *BRIP1* | *CREBBP* | *FANCI* | *FGFR2* | *JAK3* | *MSH3* | *PALB2* | *RAD51D* | *TSC1* |
| *AKT2* | *BTK* | *CSF1R* | *FANCL* | *FGFR3* | *KDR* | *MSH6* | *PDGFRA* | *RAD54L* | *TSC2* |
| *AKT3* | *CARD11* | *CTNNB1* | *FBXW7* | *FGFR4* | *KIT* | *MTOR* | *PDGFRB* | *RB1* | *VHL* |
| *ALK* | *CCND1* | *DDR2* | *FGF1* | *FLT1* | *KMT2A(MLL)* | *MUTYH* | *PIK3CA* | *RET* | *XRCC2* |
| *APC* | *CCND2* | *DNMT3A* | *FGF2* | *FLT3* | *KRAS* | *MYC* | *PIK3CB* | *RICTOR* |  |
| *AR* | *CCNE1* | *EGFR* | *FGF3* | *FOXL2* | *MAP2K1* | *MYCL1* | *PIK3CD* | *ROS1* |  |
| *ARID1A* | *CD79A* | *EP300* | *FGF4* | *GEN1* | *MAP2K2* | *MYCN* | *PIK3CG* | *RPS6KB1* |  |
| *ATM* | *CD79B* | *ERBB2* | *FGF5* | *GNA11* | *MCL1* | *MYD88* | *PIK3R1* | *SLX4* |  |
| *ATR* | *CDH1* | *ERBB3* | *FGF6* | *GNAQ* | *MDM2* | *NBN* | *PMS2* | *SMAD4* |  |
| *BAP1* | *CDK12* | *ERBB4* | *FGF7* | *GNAS* | *MDM4* | *NF1* | *PPP2R2A* | *SMARCB1* |  |
| *BARD1* | *CDK4* | *ERCC1* | *FGF8* | *HNF1A* | *MET* | *NOTCH1* | *PTCH1* | *SMO* |  |
| *BCL2* | *CDK6* | *ERCC2* | *FGF9* | *HRAS* | *MLH1* | *NOTCH2* | *PTEN* | *SRC* |  |
| *BCL6* | *CDKN2A* | *ERG* | *FGF10* | *IDH1* | *MLLT3* | *NOTCH3* | *PTPN11* | *STK11* |  |
| *BRAF* | *CEBPA* | *ESR1* | *FGF14* | *IDH2* | *MPL* | *NPM1* | *RAD51* | *TERT* |  |
| *BRCA1* | *CHEK1* | *EZH2* | *FGF23* | *INPP4B* | *MRE11A* | *NRAS* | *RAD51B* | *TET2* |  |
| *BRCA2* | *CHEK2* | *FAM175A* | *FGFR1* | *JAK2* | *MSH2* | *NRG1* | *RAD51C* | *TP53* |  |

**Table S3**. Somatic mutations detected by NGS.

|  | Patient | Gene | SNP effect | AF (%) in tumor | AF (%) in plasma |
| --- | --- | --- | --- | --- | --- |
| Tumor | 2 | FGFR1 c.1119G>T | Missense | 1.1 | 0.05914 |
|  | 2 | TP53 c.723delA | Frameshift | 39.1 | 0.07113 |
|  | 2 | RAD51C c.725A>T | Missense | 50.6 | 0.08349 |
|  | 3 | MYC c.482C>T | Missense | 9.6 | 0.5205 |
|  | 3 | NOTCH1 c.6208C>T | Missense | 5.5 | 0.3768 |
| Plasma | 2 | TP53 c.485T>A | Missense | 0.08673 | 5.4 |
|  | 3 | BRAF c.1208delA | Frameshift | 0.9126 | 1.2 |
|  | 3 | CHEK2 c.478A>G | Missense | 0.4062 | 1.7 |
|  | 3 | EP300 c.6970delG | Frameshift | 0.8962 | 1.1 |
|  | 6 | TET2 c.2218C>T | Stop-gained | 0.6923 | 1.2 |
| Tumor and Plasma | 3 | DNMT3A c.1010 C>T | Missense | 6.3 | 28.7 |
|  | 6 | TP53 c.339delA | Frameshift | 56.2 | 3.4 |
|  | 6 | EP300 c.4241A>G | Missense | 32.7 | 1.8 |

**Table S4**. Absolute and relative quantification of mutant and wild-type DNA in tumor, plasma, and saliva samples by ddPCR.

| Sample ID | Plasma MT-DNA concentration | | | Plasma WT-DNA concentration | | | FAmut(%) |
| --- | --- | --- | --- | --- | --- | --- | --- |
|  | Sample (copies/uL) | Sample corr (copies/uL) | Plasma (copias/mL) | Sample (copies/uL) | Sample corr (copies/uL) | Plasma (copias/mL) |  |
| Patient No.3  (NOTCH1 c.6208 C>T) | 0.23 | 0.21 | 2.73 | 13.1 | 11.91 | 154.83 | 1.7 |
| Patient No.6  (EP300 c.4241A>G) | 0.44 | 0.4 | 40 | 60.4 | 54.9 | 2745 | 0.72 |
| Patient No.2  (TP53 c.485T>A) | 1.12 | 1.02 | 102 | 41.9 | 38.09 | 3809 | 2.61 |

| Sample ID | Tumor MT-DNA concentration | | Tumor WT-DNA concentration | | FAmut(%) |
| --- | --- | --- | --- | --- | --- |
|  | Sample (copies/uL) | Sample corr (copies/uL) | Sample (copies/uL) | Sample corr (copies/uL) |  |
| Patient No.3  (NOTCH1 c.6208C>T) | 6.7 | 6.09 | 80.5 | 73.18 | 7.7 |
| Patient No.6  (EP300 c.4241A>G) | 29.9 | 27.18 | 69.2 | 62.9 | 30.2 |
| Patient No.2  (TP53 c.723delA) | 34.2 | 31.09 | 58.2 | 52.90 | 37 |

| Sample ID | Saliva MT-DNA concentration | | | Saliva WT-DNA concentration | | | FAmut(%) |
| --- | --- | --- | --- | --- | --- | --- | --- |
|  | Sample (copies/uL) | Sample corr (copies/uL) | Saliva (copias/mL) | Sample (copies/uL) | Sample corr (copies/uL) | Saliva (copias/mL) |  |
| Patient No.2  (TP53 c.485T>A) | 0.19 | 0.17 | 17 | 261 | 237.2 | 23720 | 0.07 |

*Abbreviations:* MT-DNA, mutant DNA; WT-DNA, wild-type DNA; FAmut, fractional abundance of mutant copies.
